# A Novel Expansin Protein from the White-Rot Fungus *Schizophyllum commune*

**DOI:** 10.1371/journal.pone.0122296

**Published:** 2015-03-24

**Authors:** Omar Eduardo Tovar-Herrera, Ramón Alberto Batista-García, María del Rayo Sánchez-Carbente, María Magdalena Iracheta-Cárdenas, Katiushka Arévalo-Niño, Jorge Luis Folch-Mallol

**Affiliations:** 1 Instituto de Biotecnología. Facultad de Ciencias Biológicas, Universidad Autónoma de Nuevo León, San Nicolás de los Garza, Nuevo León, México; 2 Centro de Investigación en Biotecnología, Universidad Autónoma del Estado de Morelos, Cuernavaca, Morelos, México; 3 Facultad de Ciencias, Universidad Autónoma del Estado de Morelos, Cuernavaca, Morelos, México; California State University Fullerton, UNITED STATES

## Abstract

A novel expansin protein (ScExlx1) was found, cloned and expressed from the Basidiomycete fungus *Schizophylum commune*. This protein showed the canonical features of plant expansins. ScExlx1 showed the ability to form “bubbles” in cotton fibers, reduce the size of avicel particles and enhance reducing sugar liberation from cotton fibers pretreated with the protein and then treated with cellulases. ScExlx1 was able to bind cellulose, birchwood xylan and chitin and this property was not affected by different sodium chloride concentrations. A novel property of ScExlx1 is its capacity to enhance reducing sugars (N-acetyl glucosamine) liberation from pretreated chitin and further added with chitinase, which has not been reported for any expansin or expansin-like protein. To the best of our knowledge, this is the first report of a *bona fide* fungal expansin found in a basidiomycete and we could express the bioactive protein in *Pichia pastoris*.

## Introduction

Expansins are non-enzymatic proteins that induce extensibility and stress relaxation of plant cell walls, acting as loosening agents [1,2]. Also, they are implicated in cell enlargement and other developmental events requiring cell wall loosening, such as fruit softening, seed germination and organ abscission [3].

Expansins *sensu stricto* are in the range of 225–275 amino acids and consist of two compact domains (D1 and D2) attached by a short linker region (~4 aa) [1,4,5]. D1 is distantly related to the catalytic domain of glycoside hydrolase family-45 (GH45) and D2 is distantly related to group-2 grass pollen allergens [2]. These proteins form a long shallow groove with highly conserved polar and aromatic residues suitably positioned along both domains, to potentially bind plant cell wall polysaccharides [3,6]. Furthermore, cellulose-active proteins with distant homology to either D1 or D2 only have been reported (swollenin, loosenin, cerato-platanin and group-1 allergens of grass pollen) [7–10]. Nomenclature for classifying these proteins has been established and they were designated as expansin-related proteins [5].

Many microbial expansin proteins have been reported, including BsExlx1 from *Bacillus subtilis* [4], PcExl1 from the plant pathogenic bacteria *Pectobacterium carotovorum* [6] and HcExlx2 from the marine bacteria *Hahella chejuensis* [11], with BsExlx1 being the best well characterized expansin from a non-plant source and one of the bacterial expansins crystalized to this date [4,6,11,12]. In addition, a protein designated as Asper-EXP from the plant pathogenic fungus *Aspergillus niger* was recently reported [12]. All these proteins have been demonstrated to bind and act on cellulosic networks and some of them have shown to act synergistically with cellulases and xylanases [6,11,13,14]. Although plant and bacterial expansins have been characterized in terms of polysaccharide binding profiles, filter paper weakening activity, cell wall loosening and synergistic/enhancing effect with glycosyl hydrolases as mentioned above, it is worth noting that there are no published data regarding to the effect of an expansin protein over chitin polysaccharides and no canonical expansin proteins from basidiomycete fungi have been previously reported, although expansin-related proteins have.

In this work, we identified the first true member of fungal expansin in a Basidiomycete (ScExlx1). This protein was obtained from one of the most common white-rot fungi, *Schizophyllum commune*. Also, this is the first report where an expansin protein showed a chitin hydrolysis enhancing effect in addition to being active on cellulose. Finally, a bioactive expansin from an eukaryotic non-plant source was expressed in *Pichia pastoris* for the first time.

## Materials and Methods

### Strains and growth conditions

A fungal strain of *Schizophyllum commune* RVAN10 isolated from the northeast region of Nuevo León State in Mexico was used for this work. Mycelium was grown on YPD medium (yeast extract-peptone-dextrose) for its propagation and storage. *S*. *commune* was growth (6 days and 28°C) on mineral base media [8] supplemented with 2% wheat straw as sole carbon source.

*Escherichia coli* DH5-α was used for the construction and propagation of recombinant plasmids by incubating each transformant in LB medium (Luria-Bertani, Difco, #Cat. 240230) supplemented with the appropriate antibiotics when necessary (ampicillin 100 μg/ml for pJET-ScExlx1; zeocin 25 μg/ml for pPicZαA-ScExlx1) at 37°C for 24 h. For the heterologous expression of ScExlx1 in *Pichia pastoris* all media and protocols are described in the *Pichia* expression manual (Invitrogen, #Cat. K1740-01).

### Sequence analysis: alignment, phylogenies and modeling

Proteins sequences were aligned with MUSCLE as implemented by Geneious software (Version 7.1.5. Biomatters, Ltd.) using default parameters. N-linked and O-linked glycosylation sites, disulfide bonds and the presence of signal peptide were predicted using the Hirst [15], DiANNA 1.1 [16] and the SignalP 4.1 web server [17], respectively.

Cladogram visualization of the phylogenetic trees was performed in order to describe the relationships of the expansin derived from the *S*. *commune*´s genomic analysis with other expansins and expansins-like proteins. For phylogenetic reconstruction, sequences from previously characterized/annotated in NCBI expansins/expansins-like were selected: five sequences from bacteria [4,6,11,12], four from plants [3]; gi_91806950, gi_332658441, gi_114794319 and gi_332661523) and five from fungi (*A*. *niger*, Asper-EXP [12] and other four sequences which showed the best hits during BLAST analysis using ScExlx1 sequence as query). At the same time, a radial visualization of the phylogenetic divergence was constructed in order to describe the relationship between EXLX1, EXLX2 subfamilies and ScExlx1 (in both subfamilies only those expansins experimentally evaluated were considered). Additionally, other expansins not characterized to this date and the Asper-Exp protein [12] were considered. Phylogenetic trees were prepared using the server Phylogeny.fr (http://www.phylogeny.fr/), this platform considers various bioinformatics algorithms to construct a robust phylogenetic tree from a set of sequences [18,19]; for the generation of phylogenetic trees, MUSCLE was used for the multiple alignments, Gblocks for the automatic alignment curation (in order to eliminate poorly aligned positions, not allowing smaller final blocks and less strict flanking positions), BioNJ for tree building and TreeDyn for tree drawing [19]. The Neighbor-Joining (NJ) method was used to estimate the phylogenetic tree; the aligned sequences were bootstrapped 1000 times and the Jones-Thornton-Taylor (JTT) model was used to estimate distances for amino acids [19]. The parameters used during the MUSCLE alignment were those recommended by the Phylogeny.fr platform (custom mode with 16 as the maximum number of iterations).

The amino acid sequence for the ScExlx1 expansin was submitted to the I-TASSER server [20] without constraints in order to get a three-dimensional model of the protein. A second modeling was performed using PDB 3D30 as a template. The visualization and structural alignment were obtained in VMD (Visual Molecular Dynamic).

### Cloning and heterologous expression of ScExlx1

Isolation of total RNA was performed on 6-day-old culture of *S*. *commune* on wheat straw medium using the Trizol method (Invitrogen, #Cat. 15596–026). First-strand cDNA synthesis was performed using the RevertAid H Minus First Strand cDNA Synthesis Kit (Thermo Scientific, #Cat. K1631) following the manufacturer´s instructions. The amplification of the full-length *ScExlx1* cDNA was performed using specific primers designed from *S*. *commune* H4-8 genome (http://genome.jgi.doe.gov; protein ID: 2642684): Forward primer ScExlx1F (5′-ggtaccgtccaccacaccaccgcgaa-3′) and Reverse primer ScExlx1R (5′-tctagaccgaactgcgacccgccctcg-3′). In both primers, sequences for restriction sites (underlined sequences) were added for *Kpn*I and *Xba*I recognition. The 654 bp PCR fragment was purified and cloned in the pJET vector (Thermo Scientific, #Cat. K1231) resulting in pJET-ScExlx1. The *ScExlx1* cDNA was further sequenced using the pJET primers.

The *ScExlx1* cDNA fragment cloned into pJET vector was digested with *Kpn*I and *Xba*I and purified with GeneJET Gel extraction kit (Thermo Scientific, #Cat. K0691). In parallel, pPICZαA was digested using the same restriction enzymes, and *ScExlx1* was ligated at the corresponding sites into pPICZαA in frame with both the yeast α-secretion factor and C-terminal His_6_ tag encoding sequences. pPicZαA-ScExlx1 was linearized with restriction enzyme *Sac*I and used for transformation of *P*. *pastoris* X-33 by electroporation and selected on YPD plates containing zeocin (100 μg/ml) (Invitrogen, #Cat. R250-01). The pPicZαA vector without insert was also transformed into X-33 and this strain was used as a negative control.

Ten randomly chosen Zeocin-resistant *P*. *pastoris* transformants were then screened for protein expression in 12.5 ml of BMGY (buffered complex medium containing glycerol) at 28°C in an orbital shaker (220 rpm) for 18 h to an OD_600_ of 2–6, and expression was induced by transferring cells into 50 ml of BMMY (buffered complex medium containing methanol) and growing them for a another 3 days. Each day the medium was supplemented with methanol at 0.5% (v/v). The supernatant was then analyzed by SDS-PAGE to determine which transformant had the best secretion yield.

### Protein purification and quantification. SDS-PAGE and Western Blot

Culture supernatant was concentrated using Vivaspin centrifugal units (Sartorius, #Cat. VS2001) with a 10 kDa cut-off at 7,000 rpm and 4°C. The concentrated supernatant was loaded on a HisTrap excel Nickel column (GE Healtcare, #Cat. 17-3712-05) connected to a peristaltic pump and previously equilibrated with phosphate buffer (20 mM NaH_2_PO_4_, 20 mM Imidazole, 0.5 M NaCl pH 7.4). SDS-PAGE and Western Blot analyzed fractions were pooled, buffer exchanged and dialyzed using Vivaspin centrifugal units coupled to diafiltration cups (Sartorius, #Cat. VSA005) against acetate buffer (50 mM, pH 5).

Total protein concentrations of crude supernatant or purified fractions were determined by Bradford assay [21] using Bovine Serum Albumin (BSA) as calibration standard. Molecular mass estimation of recombinant ScExlx1 was done loading 5 μg of protein into 12% SDS-polyacrilamyde gel. Protein bands were visualized by Coomassie Blue R-250 (Sigma-Aldrich) staining and PageRuler Plus Pre-stained Protein Ladder (Thermo Scientific, #Cat. 26619) was used for molecular mass estimation.

For Western blot analysis, purified ScExlx1 was run on a 12% SDS-PAGE and blotted onto a nitrocellulose membrane (Bio-Rad) using a wet tank blotting system (Bio-Rad). After transference, the membrane was washed three times with phosphate buffer containing 0.1% Tween 20 (PBST) pH 8. The membrane was blocked with PBST plus skimmed milk (3%) for 20 min and washed with PBST. c-Myc (9E10) (Santa Cruz Biotechnology, #Cat. Sc-40) and anti-His antibodies (Roche, #Cat. 11922416001) were used for immunodetection (dilution 1:5000) and signal detection was visualized using an anti-mouse alkaline phosphatase conjugate (Sigma, #Cat. A3562) (dilution 1:10000) by incubating 30 min in PBST plus skimmed milk. The membrane was washed three times with PBST and 1 ml of Fast Red TR/Naphthol AS-MX (Sigma, #Cat. F4648) was added for detection of alkaline phosphatase. Deglycosylation was performed using PNGase F (New England Bio-labs, #Cat. P07045) in order to remove ScExlx1 N-linked glycans, according to the manufacturer´s instructions.

### Disruptive activity of ScExlx1 on cotton fibers and avicel

Cotton fibers were mercerized according to [7]; briefly, 1 mg of cotton fibers were incubated with 25% NaOH for 15 min at 4°C, and washed several times with distilled water until a pH ~7 was reached. Cotton fibers were suspended in 1 ml of sodium acetate 50 mM pH 5, containing 20 μg of ScExlx1. After incubation for 72 h at 25°C the amount of reducing sugars from supernatant was analyzed by DNS (3,5-Dinitrosalicylic acid) method as described by [22] and the fibers were washed with acetate buffer, sonicated for 1 min and visualized by light microscopy. Avicel PH-101 (1 mg) (Fluka, #Cat. 11365) was treated using 20 μg of ScExlx1 in 1 ml of sodium acetate and incubated as mentioned above. After incubation, microtubes were centrifuged at 13,000 rpm for 5 min and washed 3 times with acetate buffer. Afterwards, avicel was observed by light microscopy. Acetate buffer or proteins from mock supernatant were used as control treatments for cotton fibers and avicel experiments. For each experiment, a total of three replicates were made.

### Salt effect on binding to polysaccharides

Polysaccharides binding profile of ScExlx1 was carried out as mentioned by Chen et al. [23] with some modifications. 2.5 mg of Avicel or chitin from shrimp shells (Sigma, #Cat. C7170) were incubated with ScExlx1 (40 μg) in sodium phosphate buffer 50 mM pH 7.4 containing 0 to 500 mM NaCl for 15 min under agitation. After incubation samples were centrifuged at 13,000 rpm for 5 min at room temperature and unbound protein was measured by densitometry with Quantity One software (Bio-Rad). Bound protein quantity was determined by subtracting the amount of protein used for the experiment minus the amount of protein detected in supernatants. For each experiment, a total of three replicates were made.

### Enzymatic hydrolysis

Along with the disruptive activity of ScExlx1 experiments, an independent assay was performed under the same conditions, but adding a cellulase cocktail from *Trichoderma reesei* (Sigma, #Cat. C-2730) in order to determine if ScExlx1 treatment increased the amount of released reducing sugars during the experiments. Briefly, 0.25 U of cellulase was added to 1 mg of cotton fibers after 72 h incubation with ScExlx1 and temperature increased to 50°C for 3 h. Aliquots of 50 μl were taken at 0, 5, 10, 20, 40, 60 and 180 min. In addition to cellulolytic experiments with ScExlx1 treated cotton fibers plus cellulase, a similar experiment was performed in order to determine the effect of a chitinolytic enzyme when acting on ScExlx1 treated chitin. Briefly, chitin (5 mg) was incubated with ScExlx1 (50 or 100 μg) in phosphate buffer 100 mM pH 7.4 for 24 h at 25°C. After incubation, chitinase from *Streptomyces griseus* (0.25 U; Sigma, #Cat. C6137) was added in 1 mL reaction and incubated for 2 h at 37°C. The reactions were centrifuged at 13,000 rpm for 5 min at room temperature and 500 μl of supernatant were mixed with 500 μl of DNS and boiled for 5 min. The amount of reducing sugars released was determined by DNS method using N-acetylglucosamine (NAG) (Sigma, #Cat. A8625) as a calibration standard (1, 2, 3, 4, 5, 10, 15 and 20 μmol/ml). Phosphate buffer 100 mM pH 7.4 and BSA (50 μg) were used as controls.

### Statistical analysis

For statistical treatment of experimental data, the arithmetic mean and the standard deviation were calculated. Simple classification ANOVA tests were applied to determine significant differences between the different cases. Firstly, the assumptions of ANOVA were revised: analysis of homogeneity of variance (Hartley-Cochran-Bartlett test) and normal distribution (Kolmogorov-Smirnov and Lilliefors tests) were performed [24]. Subsequently ANOVAs were conducted to demonstrate the similarities or differences between the data of the population of samples. Finally, a post hoc analysis that defines the order of the differences found in the ANOVAs was developed. The Fisher LSD, Tukey HSD and Duncan tests were considered for the post hoc analyses [24,25]. The use of these three tests ensures greater statistical robustness of the proposed analysis. Differences were considered to be significant if p≤0.05. All statistical calculations were performed in SPSS software (Version 20).

## Results

### ScExlx1 sequence analysis

A search in the *S*. *commune* H4-8 v3.0´s genome (http://genome.jgi.doe.gov/Schco3/Schco3.home.html) to find putative expansin and expansin-like proteins was made using a *Clavibacter michiganensis* expansin sequence as query (gi|WP_012038166). Only one protein (protein ID: 2642684) of 239 amino acids with both classic domains for canonical expansins (Domain 1 from amino acid 20 to 118; Domain 2 from amino acid 123 to 239) was found. The nucleotide sequence analysis of ScExlx1 (654 bp) from *S*. *commune* RVAN10 (GenBank accession number KP698384) showed a 97.2% identity with the ScExlx1 available sequence from *S*. *commune* H4-8. The amino acid differences between them resulted in 6 amino acids changes at positions: 31 (Ser/Pro), 34 (Asn/Thr), 127 (Val/Ala), 129 (Asp/Tyr), 143 (Asp/Glu) and 225 (Ile/Val). Four of these amino acid changes (34, 127, 143 and 225) were conservative, while only changes at positions 31 and 129 were non-conservative (polar uncharged/non polar, hydrophobic; acidic polar/polar uncharged, respectively). When comparing global amino acid changes with the other two versions of *S*. *commune* genomes (*S*. *commune* Leonen D v1.0 and Tattone D v1.0, available in http://genome.jgi.doe.gov/programs/fungi/index.jsf), ten amino acid changes were detected. Changes at positions 30 (Thr/Ser), 44 (His/Arg), 170 (Ile/Val), 216 (Ser/Thr) and 221 (Thr/Ser) were conservative; while other five changes occurred as follows: 25 (Met/Thr), non polar hydrophobic/polar uncharged, 41 (Lys/Gln), polar basic/polar uncharged, 128 and 182 (both Asn/Asp) polar uncharged/polar acid, and 223 (Pro/Ser) non polar hydrophobic/polar uncharged. This comparative analysis between ScExlx1 and the available *S*. *commune* putative expansin sequences showed no discrepancies between amino acids responsible for binding to polysaccharides neither the “active” site of the protein suggested by [26]. A signal peptide (signal cleavage from Met-1 to Ala-19, score 0.811) from the ScExlx1 sequence was predicted; and we found one putative N-glycosylation site (Asn-54, score 0.99), while no O-glycosylation sites were detected.

A notably feature of plant expansins from both EXPA and EXPB families is the highly conserved formation of three disulfide bonds in D1 [3]. Similarly, putative disulfide bonds formation between positions 60–86 (score 0.011), 89–107 (score 0.0104) and 110–144 (score 0.0237) were predicted in ScExlx1. Two of the three putative disulfide bonds are located in D1 and the third is between D1 and D2, indicating a similarity between EXPB1 from *Zea mays* and ScExlx1 but was absent in BsExlx1 [3,4], being this lack of disulfide bonds a common feature in bacterial expansins.

**Fig 1:**
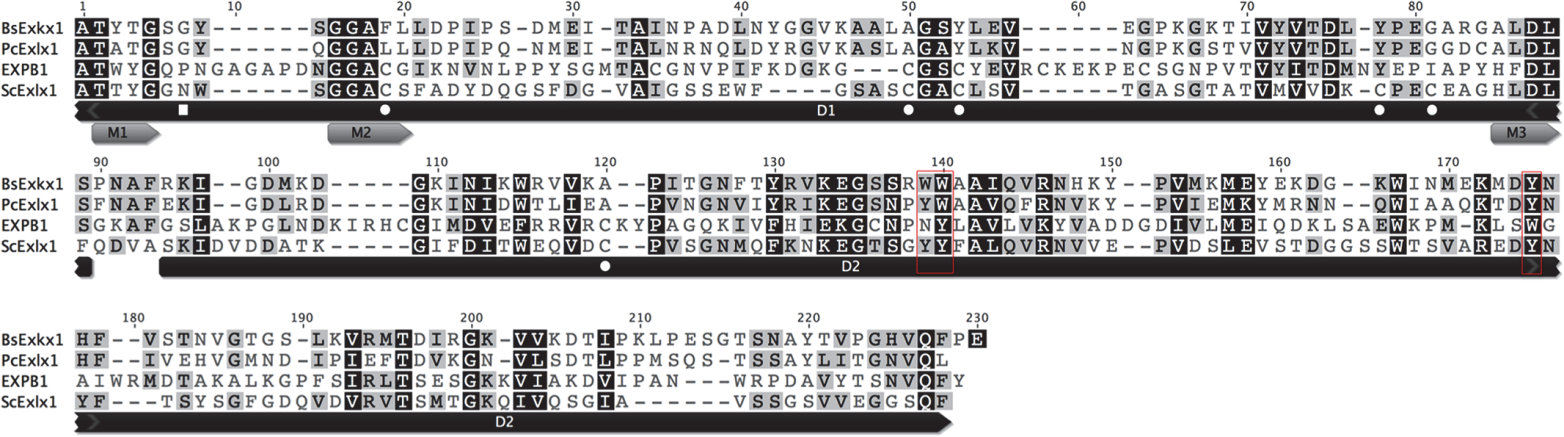
Protein alignment of BsExlx1, PcExl1, EXPB1 and ScExlx1. Darker background indicates high amino acid conservation among the sequences. M1, M2 and M3 indicate the three classic motifs of plant expansins. The boxed amino acids are important residues involved in binding and creep activity in BsExlx1. White square indicates a putative N-glycosylation site in ScExlx1 and white circles indicate the predicted residues to form disulfide bonds.

In addition, an alignment including sequences from expansins: PcExl1 [6] and two previously crystalized expansins EXPB1 [3] and BsExlx1 [4] revealed a 29.3/42.6% identity/similarity with ScExlx1 and showed that nine of the ten amino acids that form the shallow groove which potentially serves as a polysaccharide-binding site in D1, are strictly conserved between these proteins (including the most conserved residues Thr-12 and Asp-82). Similarly, the three aromatic amino acids (Trp-125, Trp-126 and Tyr-157) forming a planar platform in BsExlx1, which makes D2 resemble to type A CBM (Carbohydrate binding module) are substituted in ScExlx1 by aromatic polar/uncharged amino acids (equivalents to Tyr-161, Tyr-162 and Tyr-195). Furthermore, the three classic motifs from plant expansins (TWYG, GGACG and HFD) were conserved in ScExlx1, presenting slight modifications (T**T**YG, GGAC**S** and H**L**D) (Fig. 1).

**Fig 2:**
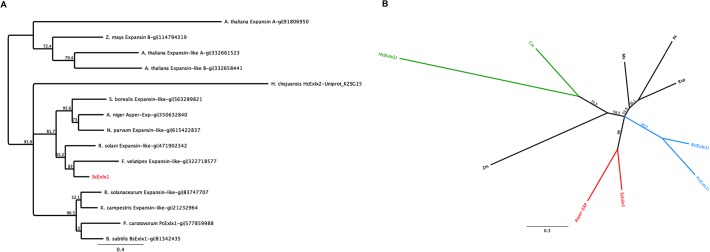
Phylogeny of ScExlx1. A) Phylogenetic relation of ScExlx1 with expansin and expansin-like proteins from plants, bacteria and fungi. Branch lengths represent the amount of genetic change between a node and its descendant. B) Phylogenetic divergence between expansin subfamilies. Ac. *Acidovorax citrulli* (gi|120612050); Mx. *Myxococcus xanthus* (gi|108762346); Rsp. *Roseiflexus* sp. (gi|148655687); Cm. *Clavibacter michiganensis* (gi|148272660). Hc. *Hahella chejuensis* (Uniprot: K2SG15) Pc. *Pectobacterium carotovorum* (gi|577859988); Bs. *Bacillus subtilis* (PDB 3D30). Asper-EXP. *Aspergillus niger* (gi|350632840).

Regarding the phylogenetic analysis, two clusters can be observed in the cladogram (Fig. 2A); one that groups the expansin proteins from plants (including members of Expansins from type A, B, expansin-like A and expansin-like B) which was used to root the tree, and a second cluster that groups previously experimentally characterized expansins [4,6,11–13] and other fungal proteins annotated as expansins in the NCBI database. The phylogenetic analysis showed that ScExlx1 is grouped directly with other fungal expansins (Ascomycota and Basidiomycota) and forming a node with a not experimentally evaluated expansin from the basidiomycete *Flammulina velutipes* (an endophytic fungus). A second sub-branch with prokaryotic expansin-like proteins is observed in Fig. 2A. It is important to note that the HcExlx2 expansin is forming a separate branch, showing no direct phylogenetic relationship with the rest of the microbial expansins.

On the other hand, a radial visualization was obtained to describe the relationship between distinct Exlx subfamilies. Lee et al. [11] proposed that Exlx expansins are divided in two different subfamilies when they found that HcExlx2 is not grouped with BsExlx1. In this order, they define a new expansin subfamily named Exlx2, but this is still controversial. The radial phylogenetic visualization revealed a markedly phylogenetic divergence between ScExlx1 with Exlx1 and Exlx2 subfamilies. This result is not surprising because ScExlx1 is a fungal protein while Exlx1 and Exlx2 are bacterial expansins. It is worth noting that the only fungal expansin previously described (Asper-Exp) groups together with ScExlx1 (Fig. 2B).

**Fig 3:**
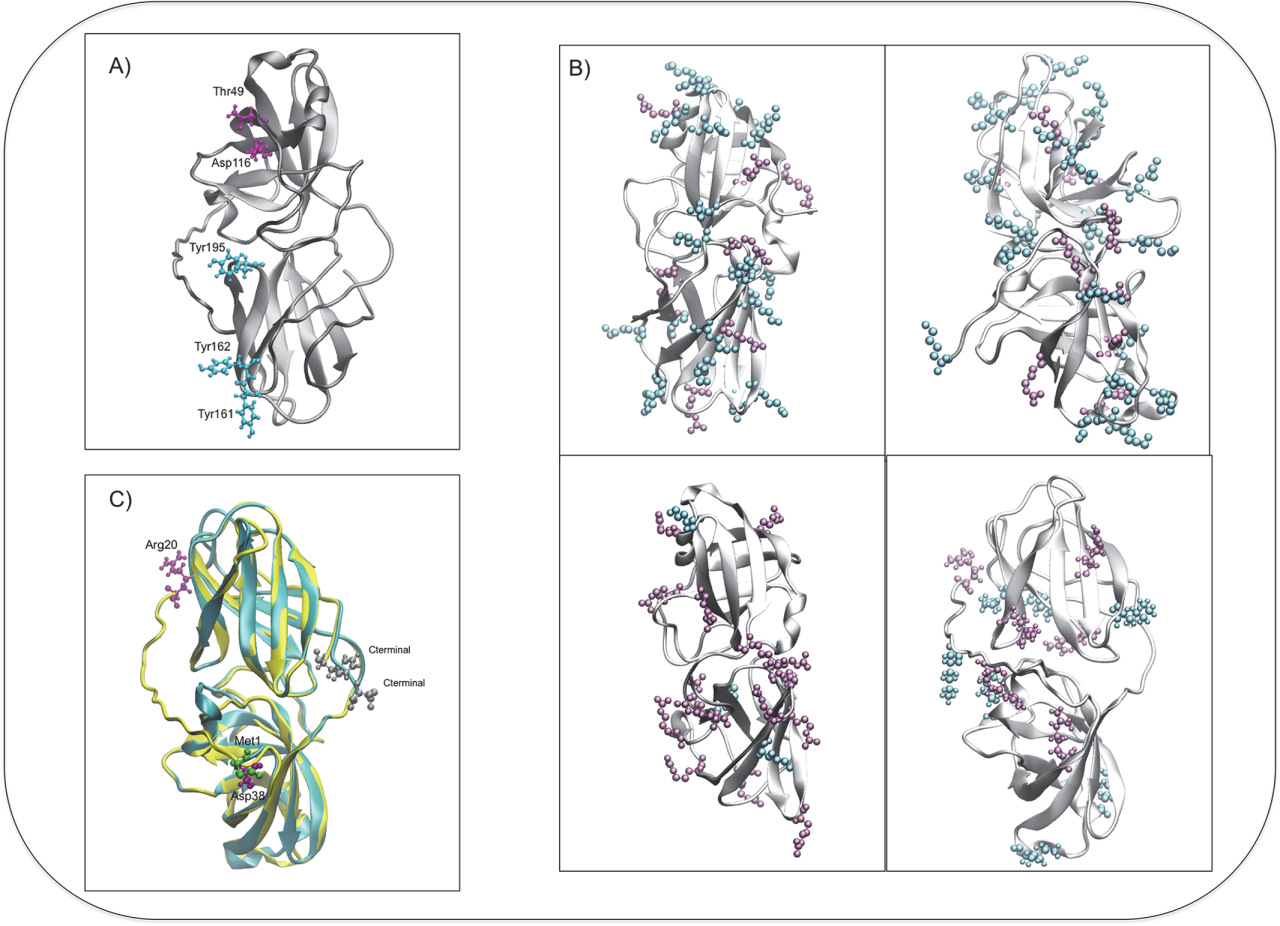
Structure of ScExlx1 and comparison with previously crystallized expansins. A) Three-dimensional model proposed for ScExlx1. Most conserved amino acids between plant and microbial expansins in D1 are depicted in magenta (Thr-49 and Asp-116). Sugar-binding residues in D2 are showed in cyan (Tyr-160, Tyr-161 and Tyr-195). B) Three-dimensional models showing the positive charged amino acids (Arg+Lys) between different EXLX proteins reported previously and ScExlx1 (Lysine is depicted in cyan and Arginine is depicted in magenta). Top-left, PDB: 3D30. Top-right, PDB: 2HCZ. Bottom-left, PDB: 4JCW. Bottom-right, ScExlx1. C) BsExlx1 (cyan model) and ScExlx1 (yellow model) superimposed, showing an N-terminal extension in ScExlx1 that it is absent in BsExlx1. Amino acids depicted in silver, C-terminal in both proteins. Amino acid in green, Met-1 of BsExlx1. Amino acids in purple, Arg-20 and Asp-38 depicting the N-terminal extension in ScExlx1).

In our first attempt to model ScExlx1, I-TASSER selected as templates expansins from the PDB: 3D30 (BsExlx1 from *Bacillus subtilis*), 2HCZ (EXPA from *Zea mays*) and, 4JCW and 4JJC (both Exlx from *Clavibacter michiganensis*). A fifth PDB was chosen to model ScExlx1, 1N10 (a crystal structure from *Phleum pratense*, a major timothy grass pollen allergen). A three-dimensional model beginning in Arg-20 (the signal peptide was removed before modeling) with TM-score of 0.73±0.10 and C-score of 0.14 was obtained from I-TASSER. In this attempt PDB 3D30 was identified by I-TASSER as the major template. In order to obtain a more accurate three-dimensional model, we submitted a new modeling round using PDB 3D30 as a template and obtained a definitive model (Fig. 3A) with TM-score of 0.8±0.10 and C-score of 0.49. These score values confirm a high confidence in the quality of the obtained model and additionally suggest the expansin activity of the amino acidic sequence ID: 2642684 from *S*. *commune*.

As it has been described for PcExlx1, we also found a marked difference in the number of positively charged residues (Arg+Lys) in BsExlx1 compared with ScExlx1. Fig. 3B shows the amount of these residues in the crystallized expansins: BsExlx1 (27 residues), EXPB (26 residues) and *Clavibacter* Exlx (19 residues). ScExlx1 revealed only 13 (Arg+Lys), even less than PcExlx1 [16]. This characteristic supports the acidic properties of ScExlx1 (pI 4.6).

A three-dimensional superposition considering the models derived from BsExlx1 (PDB 3D30) and ScExlx1 was visualized in VMD software previous structural alignment of both models (Fig. 3C). We found an important difference in the structural alignment analysis because an extension of 37 amino acids located in the N-terminal end of ScExlx1 was observed. The function of this extension is not clear to date, it has been described in expansins from plants [3] and fungi (Asper-Exp) [12]. Due to this extension, the N-terminal end of ScExlx1 and Asper-Exp suggest a structural difference between fungal and bacterial expansins.

### Production of recombinant ScExlx1

**Fig 4:**
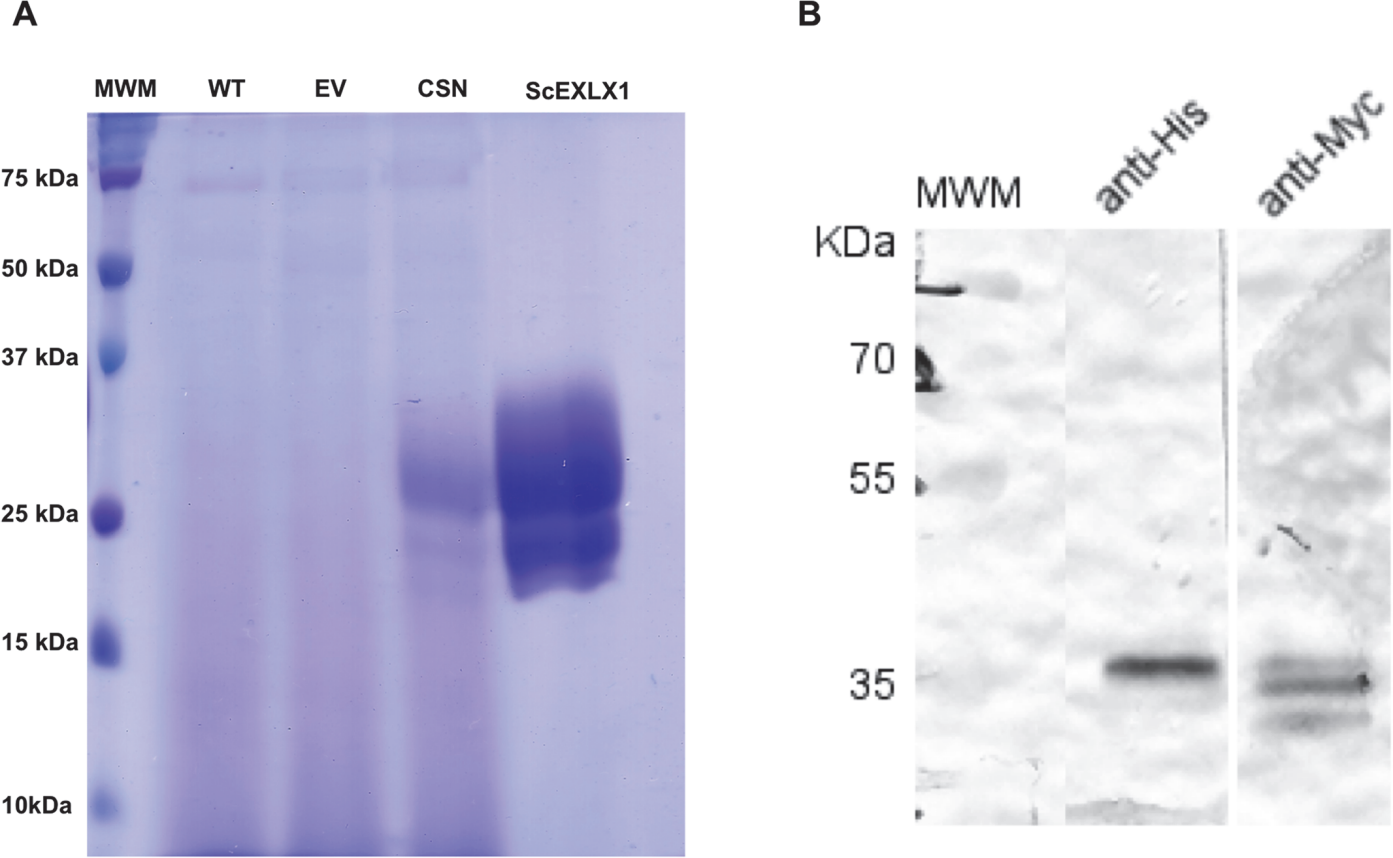
Purification and western blot analysis of recombinant ScExlx1. A) SDS-PAGE of control and recombinant strains. Supernatant from wild type *P*. *pastoris* X-33 (WT). Supernatant from *P*. *pastoris* X-33 transformed with pPICZαA empty vector (EV). Supernatant from *P*. *pastoris* transformed with pPICZαA-ScExlx1 (CSN). Purified ScExlx1. B) Western blot analysis of recombinant ScExlx1.

The ScExlx1 gene product was cloned in frame with sequences encoding the yeast α-factor secretion peptide and a (His)_6_ tag situated in C-terminus. The recombinant gene was expressed under methanol inducible AOX promoter from *P*. *pastoris*. The protein was purified and detection was carried out by SDS-PAGE in which three bands were observed with approximate molecular masses of ~24, ~28 (predicted size) and ~30 kDa (Fig. 4A). Confirmation that the three bands are ScExlx1 related was made by western blot analysis detecting the presence of the *myc* epitope present in the recombinant ScExlx1 protein (Fig. 4B).

Preliminary data suggest that the ~30 kDa band is a glycosylated form of the ScExlx1 protein, since bioinformatic analysis showed a putative glycosylation site (Asn-54) and upon treatment with PNGaseF this band disappears (data not shown).

### Effect of ScExlx1 on cotton fibers and avicel

The evaluation of the effect of ScExlx1 on mercerized cotton fibers and avicel was carried out, considering that expansins and expansin-related proteins from different sources have been demonstrated to act on cellulose [4,6,12].

**Fig 5:**
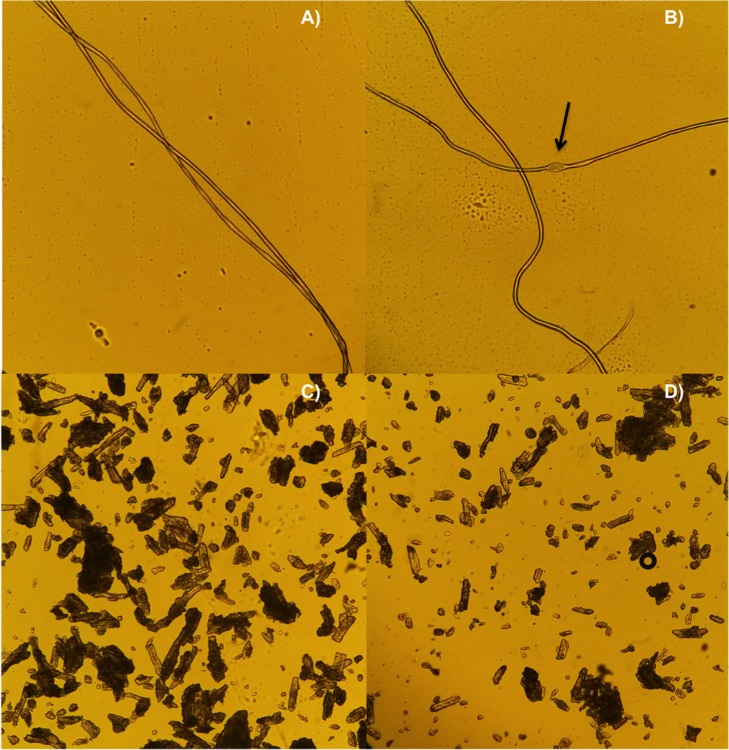
Disrupting activity of ScExlx1 on cotton fibers and avicel. Light microscopy (10X) of cotton fibers and avicel incubated with ScExlx1 or proteins from mock supernatant for 72 h at 25°C. A) Proteins from mock supernatant acting on cotton fibers. B) “Bubble” effect on cotton fibers generated by ScExlx1. C) Avicel incubated with proteins from mock supernatant. D) Reduction in avicel size particle mediated by ScExlx1.

Fibers treated with buffer or mock supernatant proteins showed no visual changes when observed by light microscopy, while fibers incubated with ScExlx1 exhibited a “bubble” effect, similar to that reported by previous works when treating cotton fibers with expansin-related proteins (Fig. 5A and 5B) [7,8]. Furthermore, when avicel was incubated with 20 μg of ScExlx1 for 72 h at 25°C a reduction in particle size was observed when compared with untreated controls (Fig. 5C and 5D). These results confirm that ScExlx1 is a functional protein and acts on crystalline cellulose.

### Binding of ScExlx1 to polysaccharides

The binding capacities of bacterial expansins (on avicel, birchwood xylan and peptidoglycan) and the salt influence in the expansin-substrate interaction have been evaluated [4,6]. In this context, we evaluated the binding capacities of ScExlx1 to avicel and birchwood xylan at different salt concentrations, and we included chitin in the binding experiments given that some expansin-related proteins have been shown to bind this polymer [8,23].

**Fig 6:**
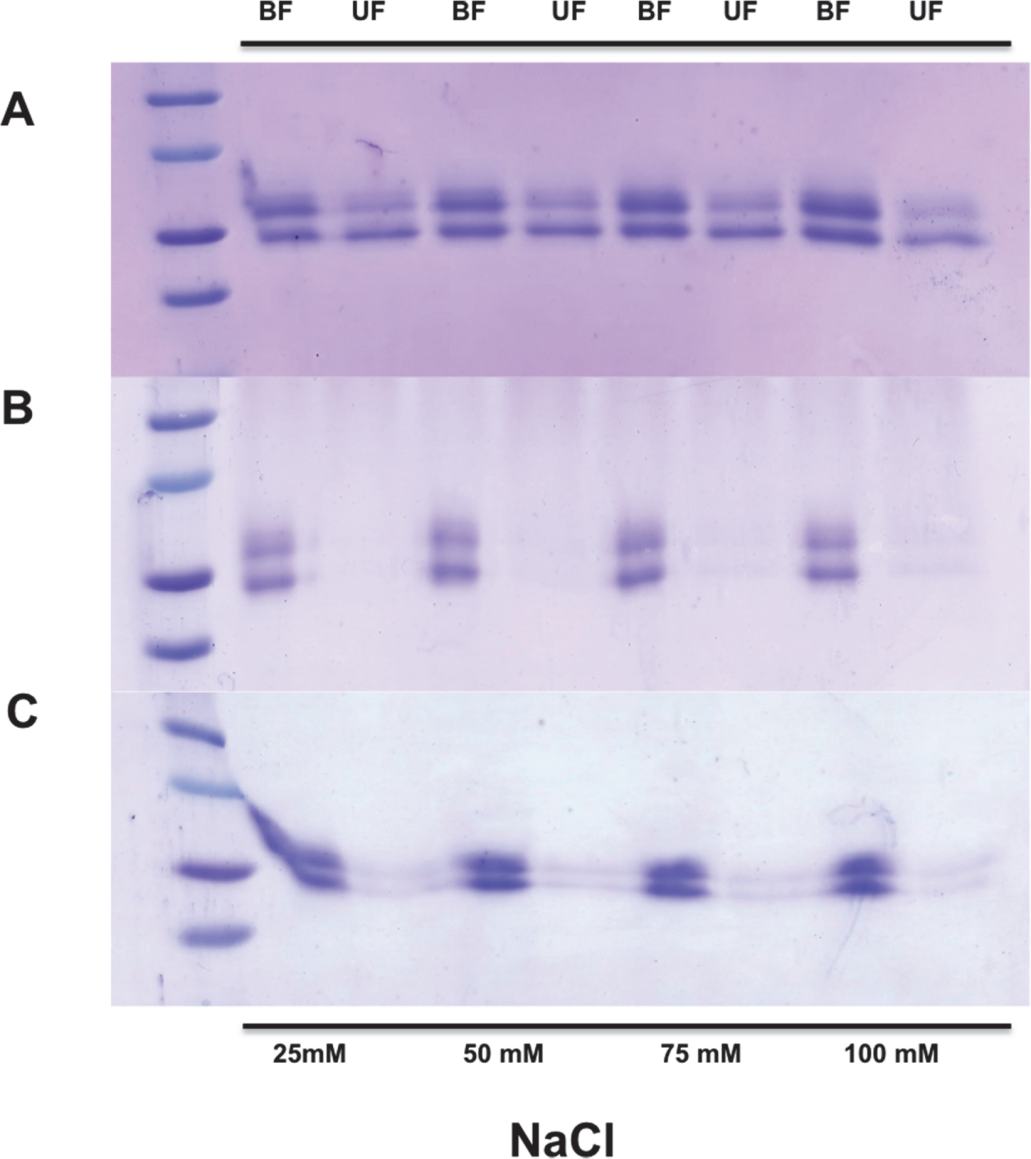
Neutral salt addition effect on ScExlx1 binding to plant cell wall polysaccharides and chitin. Nacl effect on ScExlx1 binding to: A) avicel; B) chitin; C) xylan.

ScExlx1 exhibited the ability to bind to avicel, xylan from birchwood and chitin. Nevertheless, increments in salt concentration showed no effect in binding capability of ScExlx1 to avicel under the conditions used in this study, (Fig. 6A and S1 Table), a different behavior to that reported for the bacterial expansin from *Pectobacterium carotovorum* (PcExlx1) and *Bacillus subtilis* (BsExlx1) [4,6]. In a similar manner, binding of ScExlx1 to chitin was not significantly affected when higher salt concentrations were used for the experiments (Fig. 6B and S1 Table).

Despite previous evidence of salt effect in decreasing up to 90% the binding capability of bacterial expansin from *B*. *subtilis* (BsExlx1) to xylan [27], no apparent effect was observed when increasing amounts of NaCl were assayed for this polysaccharide (Fig. 6C).

### Enzymatic hydrolysis

#### ScExlx1 and cellulase activity

**Fig 7:**
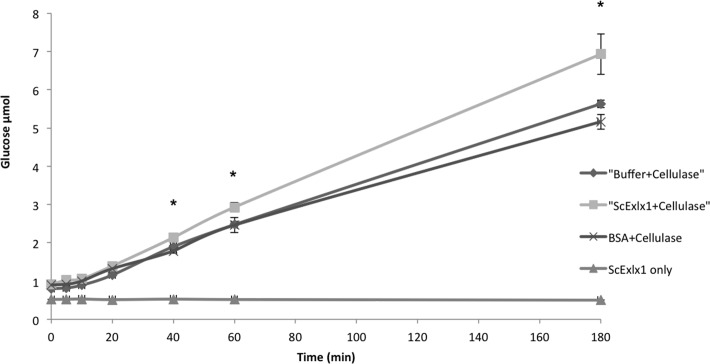
Effect of ScExlx1 on the enzymatic hydrolysis of cellulose. Mercerized cotton fibers (1 mg) were incubated with 20 μg of ScExlx1, 20 μg of BSA or sodium acetate buffer (pH 5) for 72 h at 25°C. After incubation, temperature was raised to 50°C and cellulase cocktail from *T*. *reesei* was added (0.25 U) in a 3 h experiment. Reducing sugars were quantified by DNS method and compared with a glucose standard curve. Experiments were performed in triplicate, and the data points and error bars indicate means ± standard deviations. *Statistical differences between treatments (p<0.05) at each point.

We aimed to determine if ScExlx1 produced an enhancement of cellulase activity when in combination with a *T*. *reesei* cellulase cocktail. A total of 6.9 glucose μmol were produced when cellulose hydrolysis reactions were carried out with ScExlx1 treated cotton fibers, while 5.6 glucose μmol were liberated from untreated control in a 3 h experiment (Fig. 7). The same behavior was observed when the experiment was performed for 48 h, given that 11.1 glucose μmol were produced from ScExlx1 treated cotton fibers and only 8.9 glucose μmol from untreated control (S1 Fig.). In addition, when ScExlx1 and cellulases were incubated together, no synergism between ScExlx1 and cellulase occurred (data not shown), and no reducing sugars were detected when incubating cotton fibers with ScExlx1 only.

#### ScExlx1 and chitinase activity

**Fig 8:**
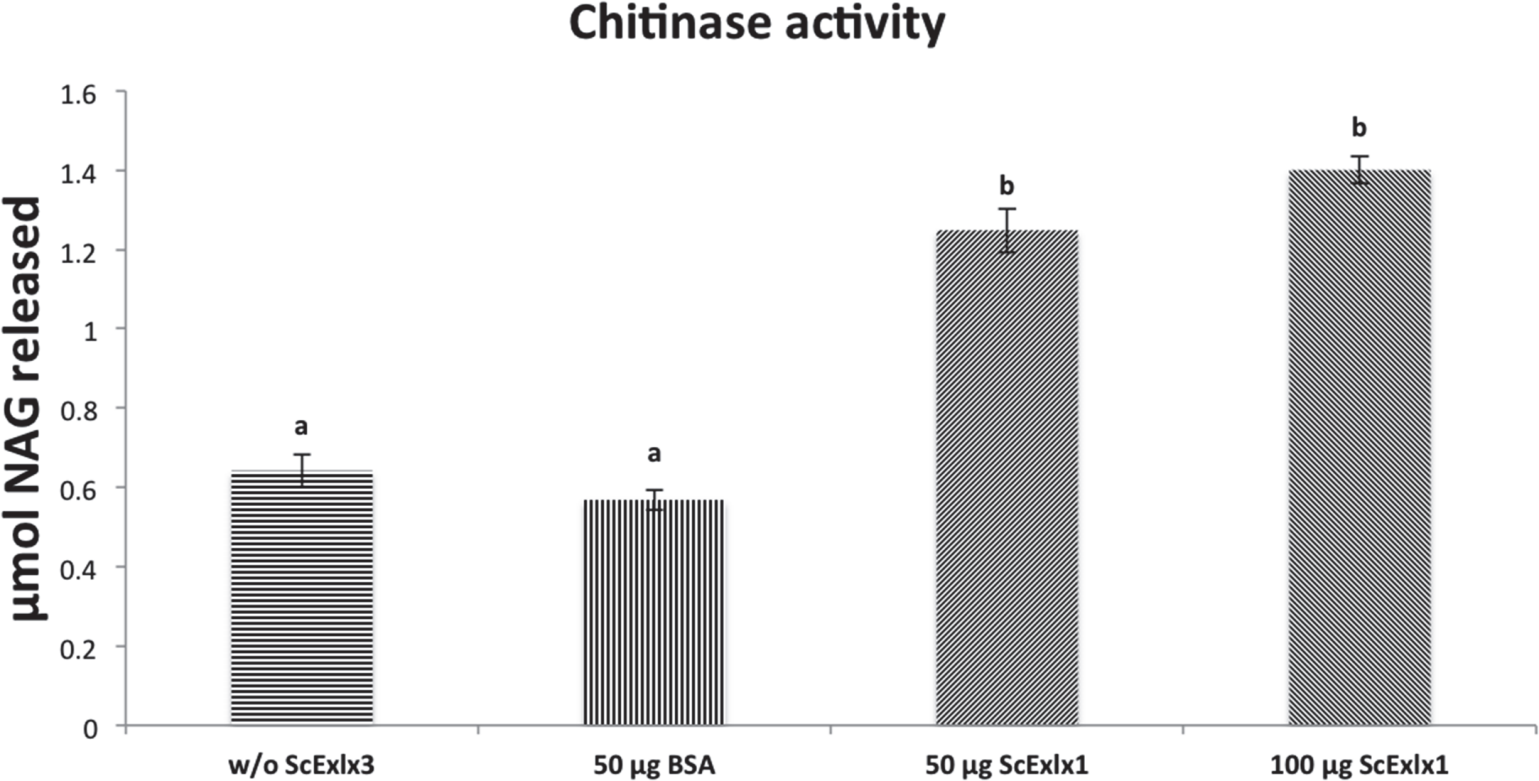
ScExlx1 is a chitin active protein that enhances chitin hydrolysis. Chitin from shrimp shells (5 mg) was incubated with 50 and 100 μg of ScExlx1, 50 μg of BSA, or sodium phosphate buffer 100 mM pH 7 for 24 h at 25°C. After incubation, temperature was increased to 37°C and chitinase from *S*. *griseus* (0.25 U) was added. After 2 hours of incubation, released N-acetylglucosamine was measured by DNS method and compared with a standard curve. Experiments were performed in triplicate, and the data points and error bars indicate means ± standard deviations. Letters indicate statistical differences in each treatment (p<0.05).

Expansin-related proteins have shown binding capacities to chitin polysaccharide [8,23]. Interestingly, no reports about the effect of an expansin or expansin-like proteins on chitin have been reported. For this reason, pretreatment of chitin with ScExlx1 before addition of chitinase from *Streptomyces griseus* was performed. This experiment resulted in releasing of 2-fold NAG amount when hydrolyzing ScExlx1 pretreated chitin when compared with buffer and BSA treated chitin (Fig. 8). Doubling ScExlx1 quantity (100 μg) during chitin pretreatment resulted in 0.2 increment of NAG μmol liberated when comparing with original concentration of ScExlx1 used for pretreatment (50 μg).

These results indicate that ScExlx1 is capable of modifying the chitin polymer, an interesting point of view given that fungal cell wall is composed mainly by this polysaccharide. Probably, expansins from fungi could play an important role during cell wall remodeling of these organisms, although more evidence to verify this function for fungal expansins is needed.

## Discussion

*Schizophyllum commune* is one of the most commonly found fungi and can be isolated from nearly all continents in the world, except for Antarctica [28]. Although *S*. *commune* has been found causing illness in humans, animals and trees, its ecological niche is to adopt a saprobic lifestyle by causing white rot [28–31]. After *S*. *commune* genome was sequenced, its enormous potential as a microbial protein factory for plant cell wall material deconstruction was revealed, and in this context we studied the protein ScExlx1 discovered trough *S*. *commune* genome analysis.

Evaluation of ScExlx1 effect on cotton fibers and avicel was studied. Previous reports have described the effect of expansin-like proteins over filter paper, avicel, PASC (Phosphoric acid-swollen cellulose) and cotton fibers [7,8,32]. As a member of the expansin superfamily, ScExlx1 caused a modification in crystalline cellulose anatomy, proposing that this effect can serve to enhance cellulose hydrolysis by *S*. *commune* when living as a white rot. In the same way, the “bubble” effect generated by loosenin [8], swollenin [7] and ScExlx1 (in this work), have been proposed to aid in the amorphogenesis step during lignocellulose degradation with hydrolytic enzymes.

An interesting finding was that sequence analysis showed a non-polar to slightly polar change in the amino acids responsible for polysaccharide binding at domain 2 (Fig. 1). This fact, did not alter the ability of ScExlx1 to bind cellulose and birchwood xylan as previously reported for plant pathogenic (PcExl1), soil (BsExlx1) and marine bacterial (HcExlx2) expansins [4,6,11]. Also, some researchers have studied the effect of NaCl over protein-protein and protein-ligand interactions [4,6,33,34]. Binding of ScExlx1 to avicel was not affected by NaCl addition even at concentrations of 0.5 M, as showed by densitometry and statistical analysis (See Fig. 6 and S1 Table), proposing that salt interaction with the ScExlx1-cellulose complex is different to that reported for other microbial expansins [4,6,11], where salt addition affects positively (PcExl1) and negatively (BsExlx1) the binding capacity of the expansin protein. With regard to the binding capability on birchwood xylan, a similar behavior to that observed with avicel was shown. It is possible that hydrophobic interactions are more important in the binding of ScExlx1 to its substrates than electrostatic bonds. Perhaps higher NaCl concentrations need to be evaluated in order to detect any effect (>0.5 M). Another possibility is that using lower substrate concentrations could allow binding changes easier to see. Additionally, HcExlx2 has the ability to bind in a stronger way to xylan from oat spelts than to cellulose [13], suggesting that bacterial expansins may have different targets in the plant cell wall architecture, since BsExlx1 binds better to whole plant cell wall and PcExl1 binds better to cellulose [4,6,35]. Besides these two plant cell wall components, we evaluated the binding capability of ScExlx1 to the fungal cell wall polysaccharide chitin, insomuch as proteins like swollenin, an expansin-like protein, loosenin containing only D1 from canonical expansins, ZmEXPB (a plant expansin), BsExlx1 and HcExlx2 have displayed better binding capacities to chitin than to cellulose. In this context, densitometry experiments showed only a small increment in bound protein quantity when comparing 0 M and 0.5 M of NaCl under the experimental conditions evaluated (S1 Table). In general, these results suggest that different interactions can be displayed by fungal and bacterial expansins depending on the polysaccharide matrix, and we can also suggest (but not conclude) that ScExlx1-polysaccharide interaction is not of ionic nature. Nonetheless, although in the case of crystalline cellulose and xylan different behaviors with regard to binding properties in the presence of NaCl are exhibited depending of the expansin evaluated, we would have expected an ionic character interaction at least between ScExlx1-chitin and ScExlx1-xylan, since amino acids Tyr-161, Tyr-162 and Tyr-195 on D2 that have been demonstrated to participate in polysaccharide binding [27] in microbial expansins could serve to contribute in the hydrogen bonding through its side chain with both polysaccharides. Perhaps, different experimental conditions need to be evaluated in order to detect a more pronounced effect. It is worth noting that in all cases both, the putative glycosylated and deglycosylated forms are able to bind the polysaccharides.

It is interesting to note that in the biotechnological field, this salt-tolerant characteristic of ScExlx1 could be useful in several ways, for example: 1) the need of novel proteins tailored for the ionic liquid process technology used for the production of advanced cellulosic biofuels, and 2) addition of acetate have shown to increase the hydrolytic capacity of some chitin deacetylases [36], so ScExlx1 could help to deacetylate the chitin polysaccharide in the presence of this enzymes in an halophile environment in order to produce chitosan, a biotechnological product with high added value. This salt-tolerant property makes ScExlx1 an ideal candidate for further research in a structure-function basis and likely industrial applications.

Regarding to enzymatic hydrolysis, expansins and expansin-like proteins have been evaluated with the aim of enhancing lignocellulose hydrolysis and reducing enzyme loadings [6,8,11,12,23,32,37–41]. ScExlx1 displayed no synergistic effect when used together cellulase cocktail from *T*. *reesei* (data not shown) what is consistent with results obtained by Olarte et al. [6] and opposite to results reported by Lee et al. [11,12]. Similarly, a slightly but significant increment in cellulose hydrolysis was detected when ScExlx1 pre-treated cotton fibers were exposed to cellulase cocktail either after 3 or 48 h. With the data obtained in this work, we demonstrated that ScExlx1 exhibits a cellulolytic enhancing effect when used previously with cellulase at low enzyme loadings.

Finally, this is the first report that shows the effect of an expansin protein on the polysaccharide chitin. This fact is very significant by two main reasons: 1) the enhancing effect of chitin hydrolysis by chitinase can be exploited in several ways as this polymer has gained huge scientific interest due to it is numerous biotechnological and medical applications [42,43]; 2) there is the possibility that ScExlx1 can be acting as a fungal cell wall remodeling factor, allowing the fungus to grow in a similar manner than that of plant expansins. Notwithstanding, it remains unclear the effect generated over chitin polymer by ScExlx1; experiments to obtain this information and analysis of physiological role of ScExlx1 in *S*. *commune* are being carried out.

## Supporting Information

S1 Fig Effect of ScExlx1 on the enzymatic hydrolysis of cellulose in a 48 h experiment. Mercerized cotton fibers (1 mg) were incubated with 20 μg of ScExlx1, 20 μg of BSA or sodium acetate buffer (pH 5) for 72 h at 25°C. After incubation, temperature was raised to 50°C and cellulase cocktail from *T*. *reesei* was added (0.25 U) in a 48 h experiment. Reducing sugars were quantified by DNS method and compared with a glucose standard curve. Experiments were performed in triplicate, and the data points and error bars indicate means ± standard deviations.
(TIF)

S1 Table Effect of NaCl over binding to polysaccharides. Experiments were performed in triplicate, and different letters indicate different statistical orders.
(DOCX)
