# Supplementary figures and images for: A Novel Expansin Protein from the White-Rot Fungus Schizophyllum commune
Source: PLoS One. 2015 Mar 24;10(3):e0122296. doi: 10.1371/journal.pone.0122296 (PMC4372547; doi:10.1371/journal.pone.0122296)

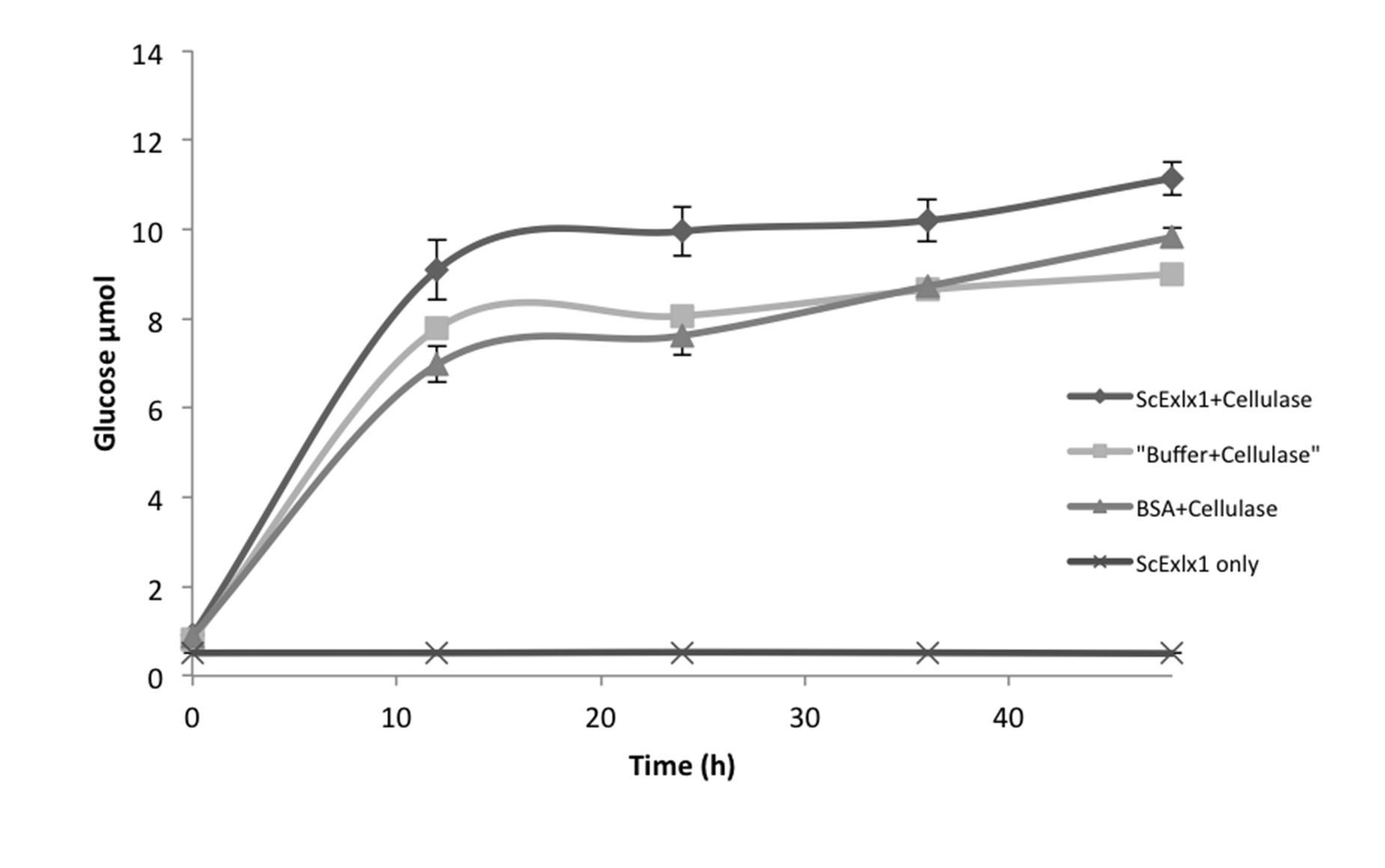

Supplement: S1 Fig — Mercerized cotton fibers (1 mg) were incubated with 20 μg of ScExlx1, 20 μg of BSA or sodium acetate buffer (pH 5) for 72 h at 25°C. After incubation, temperature was raised to 50°C and cellulase cocktail from T. reesei was added (0.25 U) in a 48 h experiment. Reducing sugars were quantified by DNS method and compared with a glucose standard curve. Experiments were performed in triplicate, and the data points and error bars indicate means ± standard deviations. (TIF) [file pone.0122296.s001.tif]
