# Supplementary material for: A Novel Expansin Protein from the White-Rot Fungus Schizophyllum commune
Source: PLoS One. 2015 Mar 24;10(3):e0122296. doi: 10.1371/journal.pone.0122296 (PMC4372547; doi:10.1371/journal.pone.0122296)
Supplement: S1 Table — Experiments were performed in triplicate, and different letters indicate different statistical orders. (DOCX) [file pone.0122296.s002.docx]

| **Bound protein (μg)** | | |
| --- | --- | --- |
| **NaCl (mM)** | **Avicel** | **Chitin** |
| 0 | 33.07 ± 0.59^a^ | 29.62 ± 1.20^a^ |
| 100 | 33.60 ± 0.25^a^ | 31.36 ± 0.80^ª,b^ |
| 200 | 35.15 ± 0.99ª^,b^ | 32.96 ± 2.02^b^ |
| 350 | 35.45 ± 0.08^ª,b^ | 33.72 ± 0.05^b,c^ |
| 500 | 36.78 ± 0.76^b^ | 36.41 ± 0.76^c^ |

**Table 1**. Binding of ScExlx1 to avicel and chitin at different salt concentrations.

Different letters indicate different statistical orders.
